# The Application of Duck Embryonic Fibroblasts CCL-141 as a Cell Model for Adipogenesis

**DOI:** 10.3390/ani14202973

**Published:** 2024-10-15

**Authors:** Dan-Dan Sun, Xiao-Qin Li, Yong-Tong Liu, Meng-Qi Ge, Zhuo-Cheng Hou

**Affiliations:** National Engineering Laboratory for Animal Breeding, Key Laboratory of Animal Genetics, Breeding and Reproduction of the Ministry of Agriculture, College of Animal Science and Technology, China Agricultural University, Beijing 100193, China; sundandan@cau.edu.cn (D.-D.S.); lixiaoqin@alu.cau.edu.cn (X.-Q.L.); lyt@cau.edu.cn (Y.-T.L.); gemq04@cau.edu.cn (M.-Q.G.)

**Keywords:** duck, adipogenesis, CCL-141, cell model, gene knockout

## Abstract

**Simple Summary:**

This study demonstrates that the duck embryo fibroblast cell line CCL-141 can undergo adipogenesis, vital for understanding fat cell development in ducks. Treatments with chicken serum, fatty acids, insulin, and all-trans retinoic acid induced fat cell formation, as evidenced by Oil Red O staining and a gene expression analysis. Moreover, the CRISPR/Cas9 knockout of the adipogenesis gene *PPARγ* in CCL-141 cells confirmed the cell line’s utility for studying adipogenesis-related gene functions. These findings validate CCL-141 as a model for adipogenesis research, which will aid in uncovering its regulatory mechanisms.

**Abstract:**

The duck embryo fibroblast cell line CCL-141, which is currently the only commercialized duck cell line, has been underexplored in adipogenesis research. (1) Background: This study establishes an experimental protocol to induce adipogenesis in CCL-141 cells, addressing the importance of understanding gene functions in this process. (2) Methods: Chicken serum, fatty acids, insulin, and all-trans retinoic acid were used to treat CCL-141 cells, with adipogenesis confirmed by Oil Red O staining and gene expression quantification. CRISPR/Cas9 technology was applied to knockout *PPARγ*, and the resulting adipogenic phenotype was assessed. (3) Results: The treatments promoted adipogenesis, and the knockout of *PPARγ* validated the cell line’s utility for gene function studies. (4) Conclusions: CCL-141 cells are a suitable model for investigating duck adipogenesis, contributing to the understanding of regulatory factors in this biological process.

## 1. Introduction

Poultry meat is an important source of human nutrition, providing a variety of essential nutrients. In addition to being a necessary energy source, its fats contain significant amounts of fat-soluble vitamins and fatty acids [1,2,3], which are crucial for human health, growth, and development. Furthermore, the fat content significantly influences the taste and quality of meat [4]. Consequently, altering the lipid deposition can impact meat quality and production [5]. Adipocytes, the primary cell type responsible for lipid deposition [6], are derived from mesenchymal stem cells [7]. This process is known as adipogenesis. Adipogenesis is a complex process involving a series of regulatory factors, including adipogenic transcription factors, cytokines, and hormones [8]. Adipogenesis is comprehensively regulated by multiple genes [9]. For instance, peroxisome proliferator-activated receptor gamma (*PPARγ*), CCAAT/enhancer binding protein beta (*C/EBPB*), CD36 molecule (*CD36*), fatty acid binding protein 4 (*FABP4*), and zinc finger protein 423 (*ZNF423*) play important roles in cell differentiation, proliferation, lipid intake, and fatty acid synthesis [5]. Among them, *PPARγ* plays a key role in adipogenesis and is considered the main regulatory gene controlling adipogenesis [10]. Despite extensive research on adipogenesis, the precise mechanism underlying it remains incompletely understood. Therefore, investigating the genetic mechanisms underlying adipogenesis is of the utmost importance for breeding desirable fat traits.

As an important economic animal, ducks have been extensively studied for their fat-related traits, including the use of genomic [11] and transcriptomic [12] research to elucidate the process of adipogenesis and identify potential candidate genes and molecular regulatory mechanisms that influence duck fat content. Most of the previous studies have used primary duck preadipocytes for experimental verification [13,14,15]. Previous studies have demonstrated that chicken serum can induce the differentiation of duck primary embryonic fibroblasts into adipocytes, and can increase lipid droplet formation in a dose-dependent manner. However, in order to investigate the role of adipogenic-related genes, a further enhancement of the induction effect is required [16]. However, due to the difficulty in isolating primary cells and their limited proliferative capacity, these cells have limitations for use in gene editing experiments, and cannot be used to further explore the transcriptional regulatory mechanisms of duck adipogenesis. Therefore, it would be of great value to establish a cell model with immortalization characteristics and the ability to undergo adipogenesis, to conduct gene editing and functional experiments to study the role of genes in adipogenesis in ducks.

Embryonic fibroblasts primarily originate from the mesoderm [17]. Embryonic fibroblast CCL-141, as the only commercially available cell line of ducks, has been widely used in the study of avian viruses and other pathogens, as well as in testing the efficacy of drugs and comparing the virulence of different virus strains [18,19]. However, there have been no reports on the use of duck embryo fibroblast CCL-141 cells to study the process of adipogenesis. Chicken embryo fibroblast DF-1 cells can be induced to differentiate into adipocytes [20], laying an important foundation for studying the adipogenesis process in birds. According to empirical data, duck meat constitutes the second-largest source of poultry meat in the marketplace. In 2023, the production of meat ducks in China surpassed 4 billion individuals, with a cumulative market value reaching CNY 126.3 billion. The consumption of duck meat accounts for one-third of the poultry meat market share. Consequently, the investigation of the adipogenic differentiation processes of ducks, as a significant category of domestic poultry, is of considerable importance [21].

Currently, some studies have utilized CRISPR/Cas9 for gene editing in ducks both in vivo and in vitro. By injecting Adeno-MSTN into primary duck embryonic cells, researchers have successfully generated heterozygous mutant offspring with a 1-base pair insertion mutation, thus providing a genome-edited duck model for further analysis [22]. Additionally, another study produced transgenic ducks that express green fluorescent protein using the CRISPR/Cas9 system [23]. The above two studies provide a technical basis for conducting in vivo editing of ducks. In the context of in vitro gene editing, gene overexpression and knockout experiments have been conducted in duck primary embryonic fibroblasts to investigate the regulatory patterns of gene expression during the viral infection process [24]. The construction of knockout plasmids targeting duck hepatitis B virus (DHBV) and their transfection into duck liver cells infected with the virus has led to the inhibition of viral proliferation within cells [25]. However, there is currently no research on the induction of adipogenesis in duck embryo fibroblast CCL-141 cells, or the feasibility of using them for gene editing experiments to investigate adipogenesis functioning.

In this study, we successfully developed a protocol for inducing adipogenesis in duck embryo fibroblast CCL-141 cells by incorporating various components into the culture medium. We found that the induction effect was better when using chicken serum, fatty acids, insulin, and all-trans retinoic acid components. Combining the expression level and Oil Red O staining, the results showed that duck embryo fibroblast CCL-141 cells could serve as a cell model for studying duck adipogenesis. By combining the effects of knocking out the key gene, *PPARγ,* of the cells, we verified the feasibility of conducting gene knockout and adipogenesis phenotype determination in the CCL-141 cell line. These results provide an experimental basis and methodological reference for further research on the molecular mechanisms of the genetic regulation of duck adipogenesis.

## 2. Materials and Methods

### 2.1. Cell Culture and Adipogenesis

Pekin duck embryo fibroblast cells (CCL-141, ATCC, #70010580, Manassas, VA, USA) were cultured in regular growth medium (Eagle’s Minimum Essential Medium, EMEM, #30-2003, ATCC, VA, USA) supplemented with 10% fetal bovine serum (FBS, #10099-141, Gibco, Victoria, Australia) and 1% antibiotics (Penicillin–Streptomycin, #15140122, Gibco, New York, NY, USA). The cells were then trypsinized (0.25% Trypsin-EDTA, #25200072, Gibco, New York, NY, USA) and seeded into 12-well plates. When they reached 80% confluence, adipogenesis was induced by replacing the regular growth medium with four different culture media: (a) EMEM with 10% FBS as the control; (b) EMEM with 10% chicken serum (**CS**, #SBJ-SE-C011, Sbjbio, Beijing, China); (c) EMEM with 10% CS, 1:100 fatty acids (#L9655, Sigma-Aldrich, St. Louis, MO, USA, linoleic acid and oleic acid, 2 mol/mol albumin each, 100 mg/mL BSA), and 10 μg/mL insulin (#PB180432, Procell, Wuhan, China); and (d) EMEM with 10% CS, 1:100 fatty acids (#L9655, Sigma-Aldrich, St. Louis, MO, USA), linoleic acid and oleic acid, 2 mol/mol albumin each, 100 mg/mL BSA), 10 μg/mL insulin, and 40 μg/mL all-trans retinoic acid (atRA, #ST1627, Beyotime, Beijing, China). After 48 h of adipogenesis, 500 μL of fresh medium with the respective components was added to each well and incubated for an additional 24 h.

### 2.2. Oil Red O Staining and Lipid Droplet Analysis

To quantify the lipid droplet content after 72 h of adipogenesis, the cells were fixed with 10% neutral formalin fixative (#Top0372, Biotopped, Beijing, China) for 1 h, stained with Oil Red O (#C0158M, Beyotime, Beijing, China), and observed and photographed under a microscope. The stained cells were washed with 100% isopropanol and the accumulated lipid droplets were quantified using a NanoDrop 2000C spectrophotometer (Thermo Fisher Scientific, San Jose, CA, USA) at an absorbance of 510 nm. The experiment was performed with 3 biological replicates and 3 technical replicates.

### 2.3. Gene Expression Analysis

The total RNA was extracted from the cells following the manufacturer’s instructions (#DP451, TIANGEN, Beijing, China). The RNA was quantified using a Nanodrop 2000 spectrophotometer (Thermo Fisher Scientific, San Jose, CA, USA). Using the total RNA as the template and oligo (DT) primer, cDNA was synthesized using a reverse transcription kit (#RR047A, TAKARA). A real-time polymerase chain reaction (RT-PCR) was performed using a SYBR Green kit (#RR420A, TAKARA, Kyoto, Japan). Design specific primer pairs for a transcript using the online software primer-BLAST (https://www.ncbi.nlm.nih.gov/tools/primer-blast, accessed on 9 June 2023). We tested different annealing temperatures to optimize each pair of primers using a conventional PCR to exclude the presence of unspecific products or primer dimer synthesis; the PCR products were analyzed by 1% agarose gel electrophoresis, and the specific steps were consistent with those of a previous study [12]. The genes and primers used for the quantitative real-time PCR are listed in Table S1, including *PPARγ*, *ZNF423*, *CD36*, *C/EBPB*, *FABP4*, glycerol-3-phosphate dehydrogenase 1 (*GPD1*), diacylglycerol O-acyltransferase 2 (*DGAT2*), and perilipin 1 (*PLIN1*). Glyceraldehyde-3-phosphate dehydrogenase (*GAPDH*) was used as the housekeeping gene. The experiment was performed with 3 biological replicates and 3 technical replicates, and all the qPCR data were analyzed using the 2^−ΔΔCT^ method [26]. To confirm whether the trend of the gene expression levels related to adipogenesis before and after induction was consistent, we conducted a correlation analysis on the FPKM (>0) of duck subcutaneous preadipocytes [27], and an RT-qPCR on the data for duck embryonic fibroblast CCL-141 cells.

### 2.4. Gene Knockout

**Figure 1:**
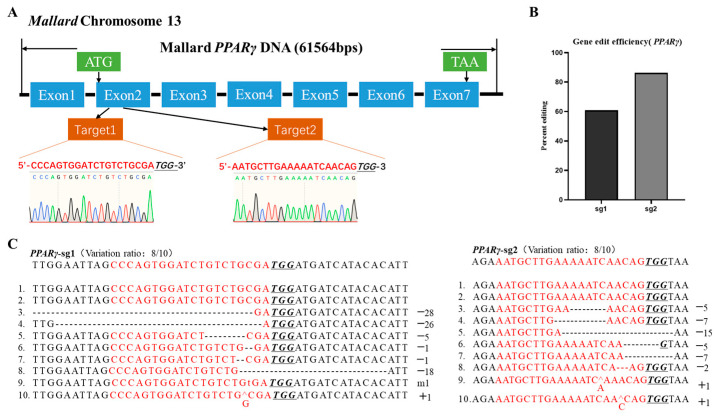
Establishment of duck *PPARγ*-knockout pooled cells using CRISPR/Cas9 system. (**A**) Structure of *PPARγ* and location of target sequence. (**B**) Analysis of knockout efficiency using website analysis based on Sanger sequencing peak graph. (**C**) Analysis of *PPARγ* knockout efficiency using single cloning. Red font corresponds to target sequence. Deleted nucleotides are marked with dashes, inserted nucleotides are represented with caret “^”, mutational nucleotides are represented with lowercase letters, and protospacer adjacent motif (PAM) sequence is indicated with italics and underlined.

According to the principles of CRISPR/Cas9 design [28], we designed two target small guide RNAs (sgRNAs) (sg1 and sg2) targeting the second exon of the *PPARγ* mRNA in the duck genome (Figure 1A) using online software http://crispr.dfci.harvard.edu/SSC/ (accessed on 9 July 2023) [29]. The PX459 plasmid contained the scaffold and Cas9 expression cassette, and the target fragments with sticky ends corresponding to the sg1 and sg2 target sequences were obtained by the direct annealing of two oligonucleotides (Table S2). The annealed fragments were then cloned into the PX459 plasmid. After DNA sequencing validation, the sgRNA plasmids were named *PPARγ*-sg1 and *PPARγ*-sg2.

### 2.5. Plasmid Transfection and Editing Efficiency Identification

The CCL-141 cells were passaged every three days at a split ratio of 1:2, plated at 8 × 10^4^ cells per well in 12-well plates 12 h before transfection. The plasmids were extracted through a reagent kit (DP118, Tiangen, Beijing, China). Each well was transfected with 1500 ng of plasmids using a constant 4.5 μL fugene (E2311, Promega, Fitchburg, WI, USA), and selected with puromycin (0.75 ug/mL) for 24 h. Afterward, an ordinary culture medium was used for cultivation. When the cell density increased to over 80%, some cells were taken for DNA identification (DP304, Tiangen, Beijing, China), and the remaining cells continued to be cultured. The target fragments were amplified from the genomic DNA of the cells using specific primers (Supplementary Table S2), and the amplified fragments were recovered using a recovery kit (#D2110-02, Magen, Beijing, China) and eluted with ddH_2_O. The recovered products were subjected to DNA sequencing and, in addition, the recovered products were cloned into a pEASY-blunt3 vector (#CB301, TransGen Biotech, Beijing, China). The sequencing process was performed by Beijing Ruibo Xingke Biotechnology Co., Ltd. (Beijing, China), and the alignment of the DNA of the recovered products with the target genes was analyzed using https://tide.nki.nl/ (accessed on 10 August 2023) [30]. Combining the peak plots of the unedited cells and pooled cells, the website analyzed the nest peak distribution in the target region of *PPARγ* to obtain the knockout efficiency (Figure S3). In addition, to analyze the off-target efficiency of the gene edit in the CCL-141 cells (Table S3), we used the online prediction tools http://crispor.tefor.net/crispor.py (accessed on 21 August 2023) [31] and https://cctop.cos.uni-heidelberg.de:8043/index.html (accessed on 21 August 2023) [32] to analyze the potential off-target sites. Six predicted gene fragments were selected for amplification and cloned into a pEASY-blunt3 vector for sequencing to detect the off-target efficiency.

### 2.6. Pooled Cells with Knocked-Out PPARγ Identification

After sequencing confirmation, the cells transfected with *PPARγ*-sg2 were expanded and cultured in a regular growth medium to obtain enough pooled cells. Before conducting the phenotype validation, some cells were taken for DNA identification (DP304, Tiangen, Beijing, China), and determined the knockout efficiency (84.8%) using the nested peak sequencing method shown. The wild-type cell served as the control group. Both the wild-type cells and experimental group cells were cultured in a conventional growth medium before induction. During induction, they were cultured in a medium (d) containing chicken serum, oleic acid, linoleic acid, insulin, and atRA to induce adipocytes.

### 2.7. Western Blot Analysis

The wild-type and pooled cells in a 6-well plate were washed with PBS 3 times, and the cell lysates were prepared after lysing the cells with a lysis buffer containing Phenylmethanesulfonyl fluoride (PMSF, PI101, Genesand, Beijing, China) and the whole process was carried out on ice. The proteins in the sample were separated by SDS-PAGE (P0015F, Beyotime, Beijing, China) and followed by blotting onto a PVDF membrane (FFP32, Beyotime, Beijing, China). The membrane was incubated with *PPARγ*-specific antibodies (ABclonal, A11183, Wuhan, China) overnight at 4 °C; *β-Actin* was the internal reference gene (Proteintech, 20536-1-AP, Wuhan, China). Then, it was co-incubated with a secondary antibody (Proteintech, SA00001-2, Wuhan, China) at room temperature for 1 h, and chemiluminescence detection reagents were used to observe the signal bands. We analyzed the band intensity using ImageJ and performed a t-test (3 biological replicates and 3 technical replicates).

### 2.8. Cell Counting Kit-8

To test the effect of *PPARγ* gene knockout on the proliferation of the CCL-141 cells, we used a CCK-8 cell counting kit-8 (#CK04-500T, Dojindo, Tabaru, Japan) to evaluate the proliferation of the CCL-141 cells. Wild-type and *PPARγ*-KO cells were seeded in 96-well plates and subjected to CCK-8 assays at different time points after seeding (0 h, 24 h, 48 h, 72 h, and 96 h). To each well was added 10% CCK-8 solution diluted in the culture medium and incubated at 37 °C for 1.5 h. The absorbance at 450 nm was measured using a spectrophotometer (Thermo Fisher Scientific, San Jose, CA, USA). The experiment was performed with 3 biological replicates and 3 technical replicates. We established a standard curve for the CCK-8 assay through different cell number gradients and their respective absorbance (O.D. values) (Figure S1). Based on the corresponding equation of the curve, we calculated the corresponding cell count based on the absorbance. 

### 2.9. Statistical Analysis

Unless otherwise specified, all the data are presented as the mean ± standard deviation (*n* = 3). A one-way analysis of variance was performed using SPSS software (version 26.0.0.0) to compare the means of multiple groups, and multiple comparisons were performed using Tukey’s test. A *p*-value of *p* < 0.05 was considered statistically significant.

## 3. Results

### 3.1. Induction of Adipogenesis in CCL-141 Cells by Different Culture Components

**Figure 2:**
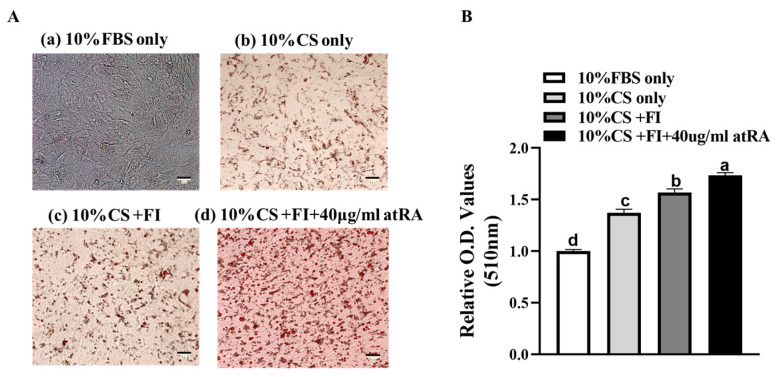
Inducing effects of different culture medium components on CCL-141 cells. (**A**) Representative images of Oil Red O staining after 72 h of induction for different culture groups: (a) EMEM with 10% FBS as control; (b) EMEM with 10% chicken serum; (c) EMEM with 10% CS, 1:100 fatty acids, and 10 ug/mL insulin; and (d) EMEM with 10% CS, 1:100 fatty acids, 10 ug/mL insulin, and 40 ug/mL all-trans retinoic acid. (**B**) Comparison of lipid droplet content in different groups extracted after Oil Red O staining (different lowercase letters on columns indicate significant differences, *p* < 0.05).

**Figure 3:**
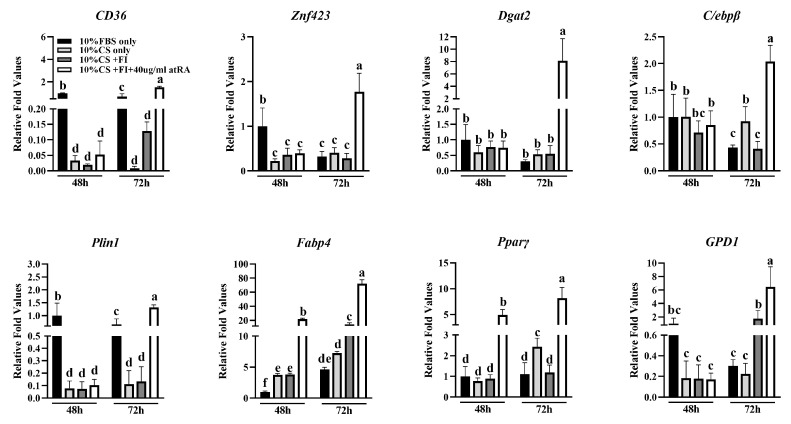
Differential expression of marker genes for adipogenesis in differentiating groups induced by culture medium containing different components for 48 h and 72 h (different lowercase letters indicate significant differences, *p* < 0.05).

This study’s findings reveal that duck embryo fibroblasts demonstrate enhanced adipogenic potential under the influence of chicken serum, oleic acid, linoleic acid, insulin, atRA, and other components. Except for the control group with fetal bovine serum, the lipid deposition content gradually increased in experimental groups b, c, and d (Figure 2A), and there were significant differences in the lipid deposition among the different groups (Figure 2B, *p* < 0.05). Experimental group d, which added atRA, had the highest lipid deposition content, indicating the strong promoting effect of atRA on adipose. Experimental group b, which added chicken serum, showed a small amount of lipid droplets, while experimental group a, which added fetal bovine serum, showed almost no lipid droplet formation. This suggests that chicken serum contains components and factors that promote adipogenesis. Compared to experimental group b, the addition of fatty acids and insulin (group c) also increased the lipid deposition content, indicating that fatty acids and insulin can enhance adipogenesis. Compared to the levels at 48 h, the expression level of the adipogenic differentiation-related genes was higher after 72 h of induction (Figure 3), which better reflects the degree of adipogenic differentiation. Therefore, we used the 72 h materials for the lipid droplet phenotypic assays.

### 3.2. Expression Levels of Adipogenic Differentiation Marker Genes in CCL-141 Cells under Different Culture Components

To characterize the adipogenesis of the CCL-141 cells, this study further measured the expression levels of adipogenesis-related genes (Figure 3). The expression levels of all the genes involved in adipogenesis were upregulated. The expression levels of four genes, including *CD36*, *C/EBPB*, *PPARγ,* and *FABP4*, were the highest after 72 h of induction (*p* < 0.05). These data indicate that supplementation with atRA, fatty acids, and insulin (FI), along with 10% CS, can induce adipogenesis and enhance the absorption and deposition of lipids in CCL-141 cells. atRA may promote lipid deposition by upregulating the expression of genes involved in lipid synthesis and accumulation. In addition, compared to the expression levels at 48 h, all the gene levels were upregulated at 72 h of induction, indicating a further enhancement of the regulatory role of CCL-141 cells in adipogenesis between 48 h and 72 h. We found that the trend of gene expression in the duck subcutaneous preadipocytes [27] and in the duck embryonic fibroblasts before and after induction was consistent (R^2^ = 0.72), with most of the marker genes showing increased expression levels compared to before induction. This indicates that CCL-141 cells can be used as a cell model for studying duck adipogenesis (Figure S2).

### 3.3. PPARγ Gene Knockout and Efficiency Identification in CCL-141 Cells

To further investigate the function of the key genes in the adipogenesis process of CCL-141 cells, this study constructed a *PPARγ* gene knockout cell model. Two targeting sequences for *PPARγ* were separately cloned into recombinant plasmids (Figure 1A, including *PPARγ*-sg1 and *PPARγ*-sg2). The plasmid containing only PX459 was used as the control. According to the peak graph results of the Sanger sequencing analysis, the knockout efficiency of sg1 and sg2 was 60.8% and 86.3% (Figure 1B and Figure S3). Furthermore, to confirm the knockout effect, the transfected cell DNA was amplified and ligated to a pEASY Blunt3 vector, and 10 single clones were selected to determine the editing efficiency (Figure 1C). The Sanger sequencing and single clone sequencing results showed that we obtained pooled cells with the knockout of the *PPARγ* gene. Based on the single clone sequencing results, we identified different editing types, including deletion, insertion, and mutation of bases, and the proportion of editing was similar to the results of the peak analysis, and no off-target effects were detected (Figure S4).

### 3.4. Inhibition of Expression of Adipogenic Marker Genes in CCL-141 Cells after PPARγ Knockout

**Figure 4:**
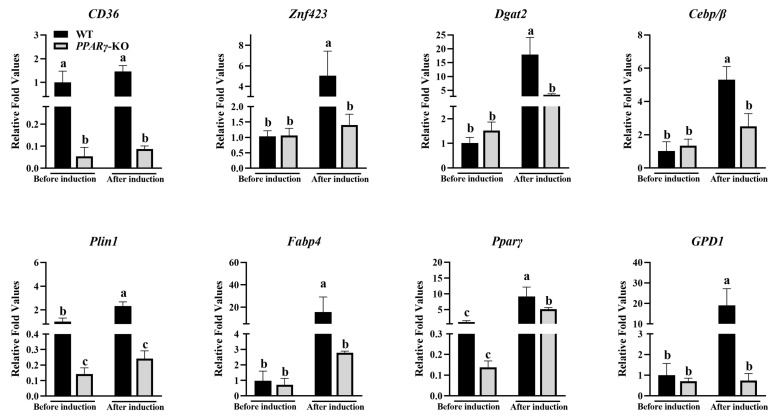
Differential expression of marker genes for adipogenesis in wild-type and knockout groups before and after 72 h of induction (different lowercase letters indicate significant differences, *p* < 0.05).

The pooled cells with *PPARγ* knockout were further cultured (edit efficiency was 84.8%, Figure S3) and induced using a medium containing chicken serum, oleic acid, linoleic acid, insulin, and atRA, with the wild-type cells as a control. To analyze the effects of *PPARγ* knockout on adipogenesis, the expression levels of the adipogenic-related marker genes were measured before and after adipogenic induction (Figure 4). We also designed qPCR primers of *PPARγ*, with upstream and downstream primers located 66 and 108 bp away from the knockout site, respectively, to detect changes in transcription levels before and after knockout. For the qPCR results, the mRNA expression level of *PPARγ* decreased compared to the wild-type cells. In addition, the results showed that the expression levels of *GPD1*, *PLIN1,* and *FABP4*, among other adipogenic marker genes, were lower in the *PPARγ* knockout group before induction. After adding the induction medium, the expression levels of all the genes in both groups further increased, but the expression levels in the knockout group were significantly lower than those in the control group (*p* < 0.05). This suggests that *PPARγ* plays a crucial role in adipogenesis, with the ability to promote the expression of other adipogenic-related genes, especially *FABP4* and *CD36*.

### 3.5. Inhibition of Adipogenesis in CCL-141 Cells after PPARγ Knockout

**Figure 5:**
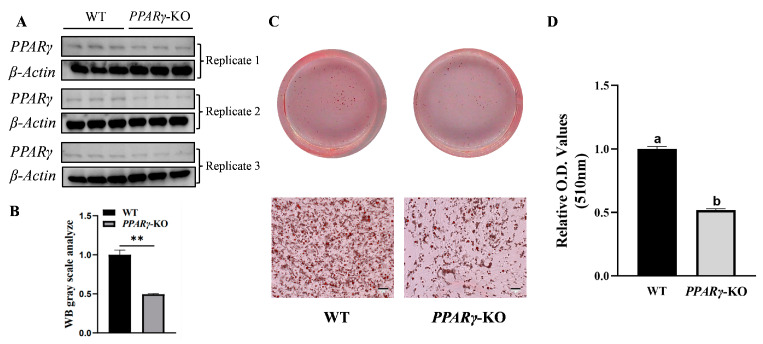
Differences in protein expression level and lipid deposition in wild-type (WT) and *PPARγ*-KO pooled cells. (**A**) Western blotting analysis of CCL-141. Protein samples of WT and *PPARγ*-KO pooled cells were extracted and Western blot analysis was performed against *PPARγ* antibody as per procedure described in “Materials and Methods” section. (**B**) Gray value analysis of protein expression level in wild-type and *PPARγ*-KO pooled cells (** *p* < 0.01). (**C**) Representative images of Oil Red O staining after 72 h of induction in wild-type and knockout groups. (**D**) Comparison of lipid droplet content in different groups extracted after Oil Red O staining (different lowercase letters indicate significant differences, *p* < 0.05).

The Western blot results clearly indicated that compared with the normal CCL-141 cells, the *PPARγ* protein expression level was decreased in the pooled cells (Figure 5A,B). The Oil Red O staining results showed that after induction, the number and size of lipid droplets in the cells with *PPARγ* knockout decreased, indicating that *PPARγ* knockout affected the synthesis and deposition of lipids (Figure 5C). Compared to the control group of wild-type cells, the relative content of lipid droplets in the knockout group decreased by approximately 50% (Figure 5D), indicating that *PPARγ* knockout inhibited adipogenesis.

### 3.6. Increased Proliferation of CCL-141 Cells after PPARγ Knockout

**Figure 6:**
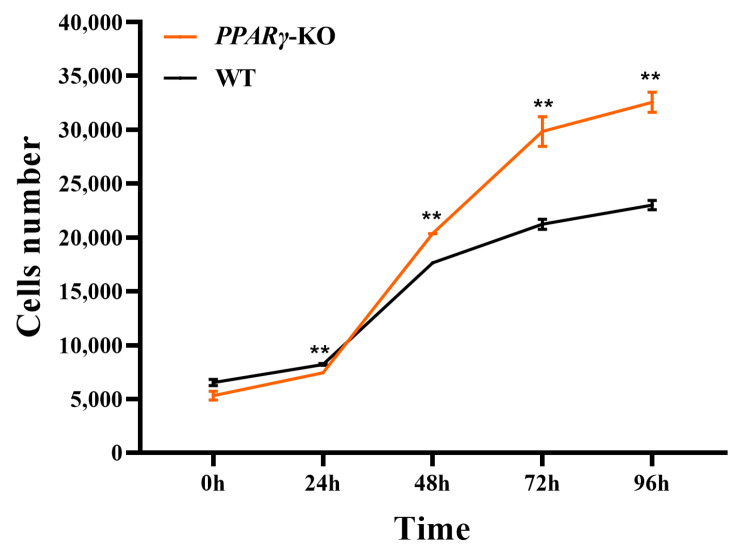
Cell proliferation curves of wild-type and knockout groups at different time points (** *p* < 0.01).

Using the same number of cells for planking at 0 d, the proliferation assay results showed that the proliferation rate of the *PPARγ*-knockout pooled cells was significantly higher than that of the wild-type cells from day 1 to day 4 after cell seeding (*p* < 0.05) (Figure 6). This result indicates that *PPARγ* can inhibit cell proliferation and, after knocking out *PPARγ*, the inhibitory effect on proliferation decreases, resulting in an increase in the cell proliferation rate.

## 4. Discussion

Currently, duck embryo fibroblasts are widely used in the fields of virology and immunity [33], but there have been limited reports on their use in gene function validation studies. As fat is an important economic trait of ducks, studying the function of duck adipogenic-related genes requires the establishment of a duck adipogenic cell model. However, the isolation of primary preadipocytes is challenging and their proliferative capacity is limited, which cannot meet the experimental requirements for gene editing and subsequent functional studies, thereby limiting the functional validation of candidate adipogenic genes in ducks. By utilizing the spontaneous immortalization characteristics of embryonic fibroblasts, the commercially available Pekin duck embryo fibroblasts CCL-141 were induced for adipogenesis, allowing for gene editing and adipogenic induction experiments to study the important functions of genes in adipogenesis. Therefore, based on the adipogenic induction of chicken embryo fibroblasts DF-1 [20], this study further confirmed the effectiveness of the adipogenic induction protocol for the duck fibroblast cell line CCL-141 through gene knockout and phenotype identification experiments. In previous studies, ducks have been utilized as models for research on muscle development [22] and viral immunity [24,25]. However, there has been no investigation into utilizing ducks as a model for lipogenesis via gene editing. The results of this study indicate that duck embryo fibroblasts can be used for gene editing and adipogenesis phenotype determination to identify the genes that regulate adipogenesis. Furthermore, embryonic fibroblasts have been shown in studies of mice and other model organisms to differentiate into osteoblasts [34] and myoblasts [35]. Subsequent research could also further explore the specific protocols for the differentiation of duck embryonic fibroblasts into osteoblasts or myocytes.

Based on the results of induction in chicken DF-1 cells [20], this study used components, such as chicken serum, oleic acid, linoleic acid, insulin, and atRA, to investigate their inducing effects on duck CCL-141 cells. The results showed that the above components could promote adipogenesis. Among them, chicken serum may provide important factors and nutrients for adipogenesis and enhance the inducing effects of other hormones [36]. Oleic acid and linoleic acid may provide sufficient energy sources for lipid deposition, increasing the uptake, storage, and utilization of triglycerides by cells [37]. Insulin [37,38,39] and atRA [40,41] may activate the key pathways of lipid metabolism, regulating adipogenesis and lipid metabolism. Consistent with previous studies, compared to the expression levels of adipogenesis marker genes after 48 h of induction, the expression levels after 72 h of induction were significantly upregulated, indicating that the inducing components can promote adipogenesis and lipid deposition. Among them, atRA is a critical inducing component, and different concentrations of atRA have different effects on cell adipogenesis. In our study, we found that 40 ug/mL was an ideal concentration of atRA for promoting adipogenesis in duck CCL-141 cells. When the concentration of atRA is too high, it inhibits the process of adipogenesis (Figure S5), which is consistent with previous research conclusions [42]. atRA regulates the metabolism and function of adipocytes by affecting the key transcription factors and signaling pathways of adipocyte differentiation [43]. Recent studies have indicated that atRA serves as a signaling molecule influencing adipogenic differentiation through the activation of nuclear retinoic acid receptors (RARs) and retinoid X receptors (RXRs) [44]. At appropriate concentrations, it can promote lipid deposition, while at higher concentrations it can inhibit the differentiation of adipocytes, reducing the number and size of adipocytes [45]. It is worth noting that different cell types may have different response concentrations to the promotion or inhibition of atRA, and further experiments with concentration gradients are needed for confirmation.

In our experimental investigations, we have observed that the efficacy of atRA inducibility is contingent upon an optimal concentration. Conversely, an excessively high concentration of this component serves to inhibit adipogenesis. Nonetheless, our studies have not yet encompassed the exploration of diverse concentration gradients of chicken serum, linoleic acid, and oleic acid, or the potential synergistic effects of different combinations of these components on adipogenesis. Such investigations could elucidate their distinct impacts on the adipogenesis potential of duck embryo fibroblasts. The utilization of rosiglitazone, dexamethasone, and 3-isobutyl-1-methylxanthine [46] has been demonstrated to induce adipogenic differentiation in embryonic fibroblasts. Furthermore, it is proposed that future research could delve into the specific protocols for the differentiation of duck embryonic fibroblasts into adipocytes, exploring the efficiency of different combinations of inducers. However, these experiments would require additional time and meticulous combination designs to be carried out.

Adipogenesis processes involve the synthesis, storage, and energy metabolism of fatty acids [47,48]. As a key transcription factor, *PPARγ* has various important effects on adipogenesis by regulating the genes related to adipogenesis [49,50,51]. In pooled cells, the mRNA expression level of *PPARγ* decreases. The insertion or deletion of bases can affect alternative splicing in transcripts [52], resulting in incorrect mRNA degradation by cells. The results of the mRNA expression levels of *PPARγ* indicate that we have indeed achieved the gene knockout of *PPARγ* in the genome. Combined with the quantitative results of the gene expression levels after induction, compared to the control group, the expression levels of the genes related to fatty acid synthesis, energy metabolism, and cell development, such as *FABP4*, *ZNF423*, and *PLIN1*, were lower in the *PPARγ* knockout group. It is possible to reduce the degree of adipogenesis by inhibiting the above biological processes. The gene expression levels before induction showed significant differences compared to the expression levels of the control group and the *PPARγ* knockout group after induction, further indicating the important role of *PPARγ* in the adipogenesis of duck embryonic fibroblasts. The interactional architecture between *PPARγ* and its associated genes is characterized by unique regulatory mechanisms. Empirical evidence has elucidated a reciprocal positive regulatory relationship between *PPARγ* and *FABP4* [53]. Furthermore, *PPARγ* operates as a proximal upstream modulator, influencing the transcriptional expression of *Plin1* [54], *CD36* [55], *DGAT2* [56], and *GPD1* [57]. Additionally, *PPARγ* is positioned downstream in the genetic cascade, responding to the regulatory signals of *ZNF423* [58] and *CEBPB* [59]. While the downregulation of these genes may be subject to additional layers of regulatory control, their reduced expression levels are indicative of a suppression of adipogenic differentiation. After *PPARγ* knockout, the proliferation rate of the cells significantly increased, consistent with the anti-proliferative function of *PPARγ* in a previous study [60].

As an important animal model, chickens can provide important references for understanding adipogenesis in poultry. However, there are still differences between different avian species, and more refined cell models are needed for further research. There are differences in the biological patterns of adipogenesis between chickens and ducks. In terms of their composition, ducks have higher intramuscular fat and polyunsaturated fatty acid contents compared to chickens [61,62]. In terms of fat distribution, duck fat is mostly distributed subcutaneously [63,64], while chicken fat is more distributed in the viscera [65,66]. These findings indicate differences in the content and distribution of adipose between chickens and ducks. Therefore, based on the induction model of chicken DF-1 cells, this study used duck embryonic fibroblasts to induce adipogenesis until they became adipocytes, which can simulate the process of adipose tissue formation and development in vitro, providing important information for comparing adipose differences between chickens and ducks.

## 5. Conclusions

In general, we developed an optimal protocol for inducing the differentiation of duck embryonic fibroblast CCL-141 cells into adipocytes by comparing the induction effects of different culture medium components. Additionally, we employed CRISPR/Cas9 to knockout *PPARγ* and conducted phenotype measurements related to adipogenesis in the knockout pooled cells. These results will provide a scheme and reference for future research on the molecular mechanisms of adipogenesis in ducks, thereby deepening the understanding of the molecular genetic mechanisms and biological functions of adipogenesis in ducks.

## Data Availability

All the results of this study are presented within the manuscript and its Supplementary Materials.

## Supplementary Materials

The following supporting information can be downloaded at: https://www.mdpi.com/article/10.3390/ani14202973/s1, Table S1: Primers designed for qPCR. Table S2: List of gRNA target sequences used in this study. Table S3: Sequences of potential off-target sites. Figure S1: Standard curve determined by CCK-8. Figure S2: Validation of RT-qPCR expression levels of adipogenesis-related genes in CCL-141. A: Correlation analysis between FPKM of adipogenic genes in subcutaneous preadipocytes of ducks and RT-qPCR data on embryonic fibroblasts of ducks. B: Histograms of FPKM of adipogenic-related genes in duck subcutaneous preadipocytes and RT-qPCR data on duck embryonic fibroblasts (* *p* < 0.05, ** *p* < 0.01). Figure S3: Detection of editing efficiency of *PPARγ*-KO using nest peak distribution analysis by EditR. A and B: Editing efficiency of *PPARγ*-sg1 and *PPARγ*-sg2. C. Editing efficiency of pooled cells in validation experiments. Figure S4: Detection of off-target efficiency of *PPARγ* using mono-clonal method. Red font corresponds to off-target sequence, and protospacer adjacent motif (PAM) sequence is indicated with italics and underlined. Figure S5: Induction effect of culture media containing different concentrations of atRA. Figure S6: Western blot images that are un-cropped and include protein marker.
