# Supplementary material for: The Application of Duck Embryonic Fibroblasts CCL-141 as a Cell Model for Adipogenesis
Source: Animals (Basel). 2024 Oct 15;14(20):2973. doi: 10.3390/ani14202973 (PMC11503743; doi:10.3390/ani14202973)
Supplement: Supplementary file 1 [file animals-14-02973-s001.zip › animals-3198584-supplementary.pdf]

animals

Article

Application of duck embryonic fibroblasts CCL-141 as a cell model for adipogenesis

Dan-Dan Sun [1], Xiao-Qin Li [1], Yong-Tong Liu [1], Meng-Qi Ge [1] and Zhuo-Cheng Hou [1,*]

[1] National Engineering Laboratory for Animal Breeding, Key Laboratory of Animal Genetics, Breeding and Reproduction of the Ministry of Agriculture, College of Animal Science and Technology, China Agricultural University, Beijing 100193, China; sundandan@cau.edu.cn (D.-D.S.); lixiaoqin@alu.cau.edu.cn (X.-Q.L.); lyt@cau.edu.cn (Y.-T.L.); gemq04@cau.edu.cn (M.-Q.G.)
* Correspondence: zchou@cau.edu.cn

Supplementary materials

Table S1. Primers designed for qPCR.

Gene (ID)	Sequence (5'-3')	PCR Product length, bp	Location
PPARγ(XM_027467226.2)	F:5′-agcccagtggatctgtctgc<br>R:5′-ctttggcaatcctggagcttgatatc	176	Exon2
ZNF423(XM_027467192.2)	F:5′-cccacagctccagcaaacc<br>R:5′-cagtctgactgaatgtctcttcacag	183	Exon5
CD36(XM_038183702.1)	F:5′-catcaaggcttgcagcaaggat<br>R:5′-gcctataaggttatctccaactgg-tatcag	167	Exon3
C/EBPB(XM_038166379.1)	F:5′-ccatgaccgagctgaccgta<br>R:5′-ctcggcgaagagatcggaaag	182	Exon1
FABP4(NM_001310375.1)	F:5′-gcccaatgtaaccatcagcataaatg<br>R:5′-tttctgtcatctgctgtggtctcat	126	Exon2
GPD1(XM_005021562.5)	F:5′-ggcttttgccaagactgggaa<br>R:5′-ggtttgccctcgtagcagatctg	177	Exon6-8

Citation: Sun, D.-D.; Li, X.-Q.; Liu, Y.-T.; Ge, M.-Q.; Hou, Z.-C. Application of duck embryonic fibroblasts CCL-141 as a cell model for adipogenesis. *Animals* **2024**, *14*, x. https://doi.org/10.3390/xxxxx

Academic Editor(s): Zissis Mamuris

Received: 24 August 2024
Revised: 14 September 2024
Accepted: 24 September 2024
Published: date

Copyright: © 2024 by the authors. Submitted for possible open access publication under the terms and conditions of the Creative Commons Attribution (CC BY) license (https://creativecommons.org/licenses/by/4.0/).

DGAT2(XM_027466264.2)	F:5′-tgcaggacttgttctggctatcc<br>R:5′-cagtcaaacaccagccaggc	184	Exon2-3
PLIN1(NM_001310391.1)	F:5′-aagaaggaggacagccacgc<br>R:5′-gtctccatggccctggacag	188	Exon2
GAPDH(XM_038180584.1)	F:5′-accactgtccatgccatcaca<br>R:5′-ccgttgagctcagggatgact	152	Exon8

Table S2. List of gRNA target sequences used in this study.

Name	Sequence (5′-3′)	Information
sg1-F	caccgCCCAGTGGATCTGTCTGCGA	gRNA sequence, 101792429, exon2
sg1-R	aaacTCGCAGACAGATCCACTGGGc	
sg2-F	caccgAATGCTTGAAAAATCAACAG	gRNA sequence, 101792429, exon2
sg2-R	aaacCTGTTGATTTTTCAAGCATTc	
F1	CCATTGTTCTTTGCCCCACAAGTTG	Sequencing, 101792429, exon2<br>F:(101792429:33937-33961),<br>551bp upstream of the edit position<br>R:(101792429:34764-34788),<br>301bp downstream of the edit position
R1	GGTGAAGGTGTGCTACGTG-TATTCC	Length of production:<br>852bp (WT and poold cells with *PPARγ*-KO*)

*The pooled cells are not missing large fragment sequences (>50bp), therefore the length of the PCR product is similar to that of the wild-type.

Table S3. The sequences of potential off-target sites.

Name	Off-target sites	Primer sequence (5′-3′)
OT-1	Intergenic<br>AgTaCTTGA-gAAATCAACAA<u>GGG</u>	F: GGCCTGGTTTAATGGTCAGC<br>R: GCAACAGCGTAAACTGCCAA
OT-2	Intergenic<br>AAaGaTa-GAAAAATCAACAG*<u>AGG</u>*	F: CTGGAACTTGTGGCTGTTGC<br>R: ATCTGGCGTTGAACAGCAGA
OT-3	Intergenic<br>AATGCacacAAAATCAACAG*<u>TGG</u>*	F: GCCTTTGGCCAGTGTTTCTG<br>R: GCTCCCATGCTGTGCTACAT
OT-4	Intron 13<br>(ENSAPLG00020009625)<br>AAaGaTa-GAAAAATCAACAG*<u>AGG</u>*	F: CCCAGGAGGCTGTGCAGT<br>R: ATGTACGATTGCAGTTTTCTGGAAGAC
OT-5	Intron 18<br>(ENSAPLG00020014420)<br>AAcaCcTG-cAAAATCAACAG*<u>TGG</u>*	F: GACTCGCCCTTGGAGCAT<br>R: CCCTTTGGCAGTTCCTTAACAAAGC
OT-6	Intergenic<br>AA-TaTTGcAAAAATCAACAG*<u>GGG</u>*	F: AGTATTTATTCAGAGTCACTGGA-GAAAAAATGT<br>R: GGAATCAAAGCCGATAA-TAATCCACAAGAGT

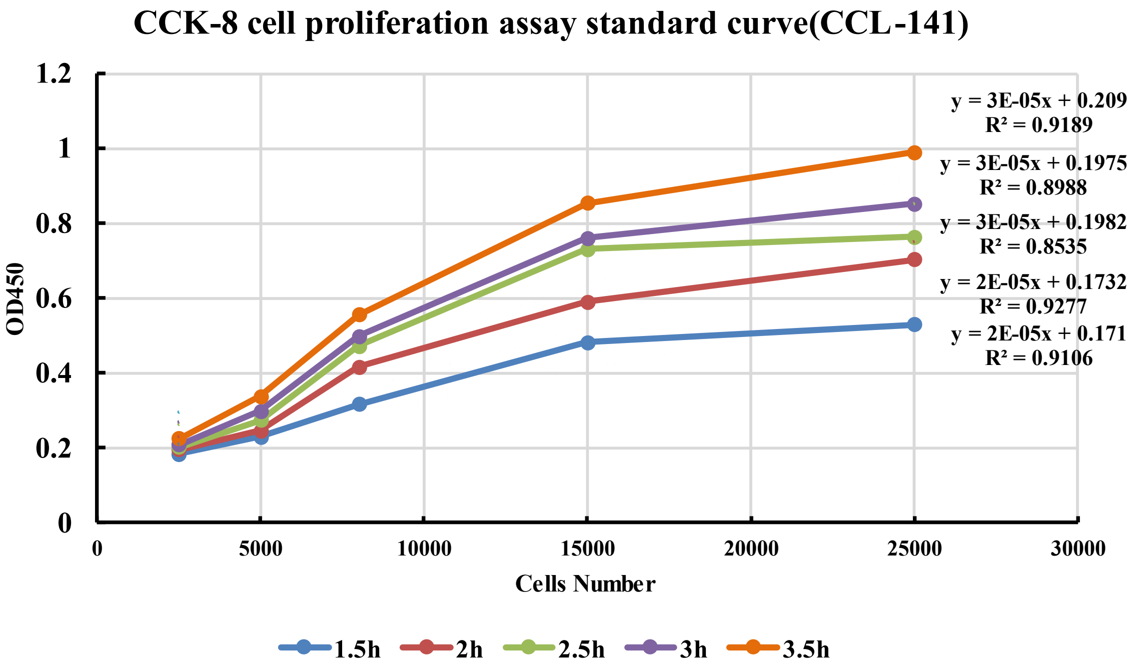

Figure S1. Standard curve determined by CCK-8.

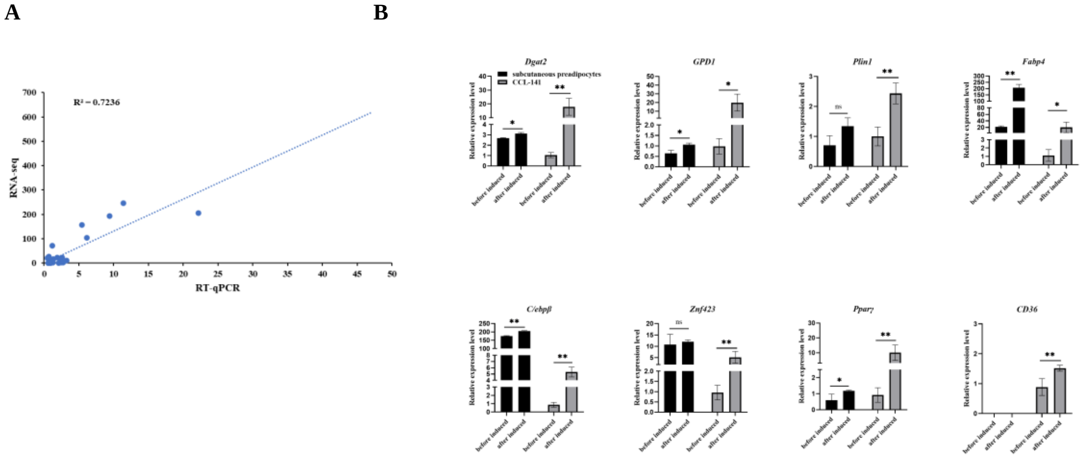

Figure S2. Validation of RT-qPCR expression levels of adipogenesis-related genes in CCL-141. A. Correlation analysis between FPKM of adipogenic genes in subcutaneous preadipocytes of ducks and RT-qPCR data in embryonic fibroblasts of ducks. B. Histograms of FPKM of adipogenic related genes in duck subcutaneous preadipocytes and RT-qPCR data of duck embryonic fibroblasts (* *P*<0.05, ** *P*<0.01).

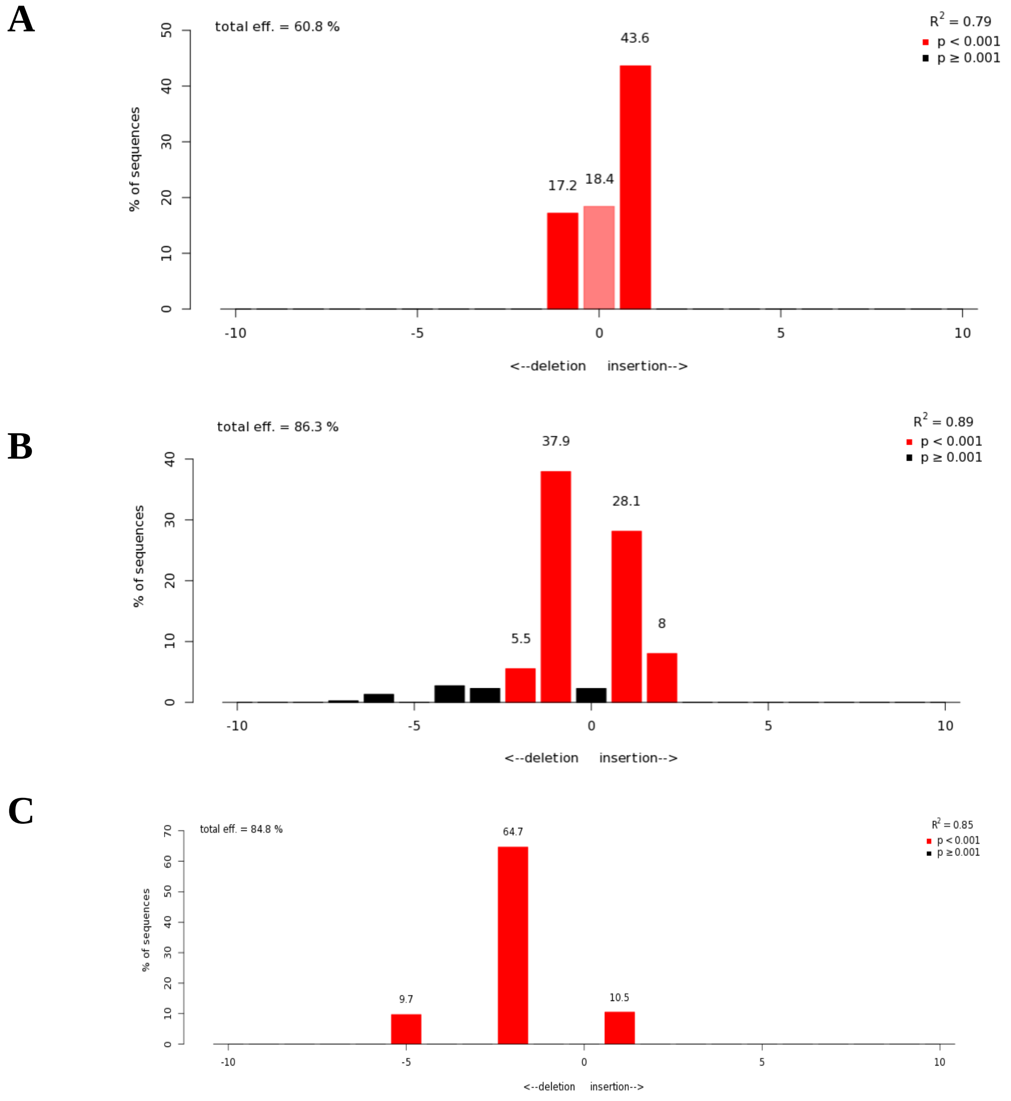

Figure S3. Detection of editing efficiency of *PPARγ*-KO using nest peak distribution analysis by EditR. A and B. Editing efficiency of *PPARγ*-sg1 and *PPARγ*-sg2. C. Editing efficiency of pooled cells in validation experiments.

OT-1

CTGAAGTACTTGAGAAATCAACAAGGG***CCC***
1. CTGAAGTACTTGAGAAATCAACAAGGG***CCC***
2. CTGAAGTACTTGAGAAATCAACAAGGG***CCC***
3. CTGAAGTACTTGAGAAATCAACAAGGG***CCC***
4. CTGAAGTACTTGAGAAATCAACAAGGG***CCC***
5. CTGAAGTACTTGAGAAATCAACAAGGG***CCC***
6. CTGAAGTACTTGAGAAATCAACAAGGG***CCC***
7. CTGAAGTACTTGAGAAATCAACAAGGG***CCC***
8. CTGAAGTACTTGAGAAATCAACAAGGG***CCC***
9. CTGAAGTACTTGAGAAATCAACAAGGG***CCC***
10. CTGAAGTACTTGAGAAATCAACAAGGG***CCC***

OT-2

GAGGAAAGATAGAAAAATCAACAG***AGG***
1. GAGGAAAGATAGAAAAATCAACAG***AGG***
2. GAGGAAAGATAGAAAAATCAACAG***AGG***
3. GAGGAAAGATAGAAAAATCAACAG***AGG***
4. GAGGAAAGATAGAAAAATCAACAG***AGG***
5. GAGGAAAGATAGAAAAATCAACAG***AGG***
6. GAGGAAAGATAGAAAAATCAACAG***AGG***
7. GAGGAAAGATAGAAAAATCAACAG***AGG***
8. GAGGAAAGATAGAAAAATCAACAG***AGG***
9. GAGGAAAGATAGAAAAATCAACAG***AGG***
10. GAGGAAAGATAGAAAAATCAACAG***AGG***

OT-3

TACAAATGCACACAAAATCAACAG***TGG***
1. TACAAATGCACACAAAATCAACAG***TGG***
2. TACAAATGCACACAAAATCAACAG***TGG***
3. TACAAATGCACACAAAATCAACAG***TGG***
4. TACAAATGCACACAAAATCAACAG***TGG***
5. TACAAATGCACACAAAATCAACAG***TGG***
6. TACAAATGCACACAAAATCAACAG***TGG***
7. TACAAATGCACACAAAATCAACAG***TGG***
8. TACAAATGCACACAAAATCAACAG***TGG***
9. TACAAATGCACACAAAATCAACAG***TGG***
10. TACAAATGCACACAAAATCAACAG***TGG***

OT-4

GAGGAAAGATAGAAAAATCAACAG***AGG***
1. GAGGAAAGATAGAAAAATCAACAG***AGG***
2. GAGGAAAGATAGAAAAATCAACAG***AGG***
3. GAGGAAAGATAGAAAAATCAACAG***AGG***
4. GAGGAAAGATAGAAAAATCAACAG***AGG***
5. GAGGAAAGATAGAAAAATCAACAG***AGG***
6. GAGGAAAGATAGAAAAATCAACAG***AGG***
7. GAGGAAAGATAGAAAAATCAACAG***AGG***
8. GAGGAAAGATAGAAAAATCAACAG***AGG***
9. GAGGAAAGATAGAAAAATCAACAG***AGG***
10. GAGGAAAGATAGAAAAATCAACAG***AGG***

OT-5

AAAAAACACCTGCAAAATCAACAG***TGG***
1. AAAAAACACCTGCAAAATCAACAG***TGG***
2. AAAAAACACCTGCAAAATCAACAG***TGG***
3. AAAAAACACCTGCAAAATCAACAG***TGG***
4. AAAAAACACCTGCAAAATCAACAG***TGG***
5. AAAAAACACCTGCAAAATCAACAG***TGG***
6. AAAAAACACCTGCAAAATCAACAG***TGG***
7. AAAAAACACCTGCAAAATCAACAG***TGG***
8. AAAAAACACCTGCAAAATCAACAG***TGG***
9. AAAAAACACCTGCAAAATCAACAG***TGG***
10. AAAAAACACCTGCAAAATCAACAG***TGG***

OT-6

TTGAAATATTGCAAAAATCAACAG***GGG***
1. TTGAAATATTGCAAAAATCAACAG***GGG***
2. TTGAAATATTGCAAAAATCAACAG***GGG***
3. TTGAAATATTGCAAAAATCAACAG***GGG***
4. TTGAAATATTGCAAAAATCAACAG***GGG***
5. TTGAAATATTGCAAAAATCAACAG***GGG***
6. TTGAAATATTGCAAAAATCAACAG***GGG***
7. TTGAAATATTGCAAAAATCAACAG***GGG***
8. TTGAAATATTGCAAAAATCAACAG***GGG***
9. TTGAAATATTGCAAAAATCAACAG***GGG***
10. TTGAAATATTGCAAAAATCAACAG***GGG***

Figure S4. Detection of off target efficiency of *PPARγ* using monoclonal method. The red font corresponds to the off-target sequence, and the protospacer adjacent motif (PAM) sequence is indicated with italics and underlined.

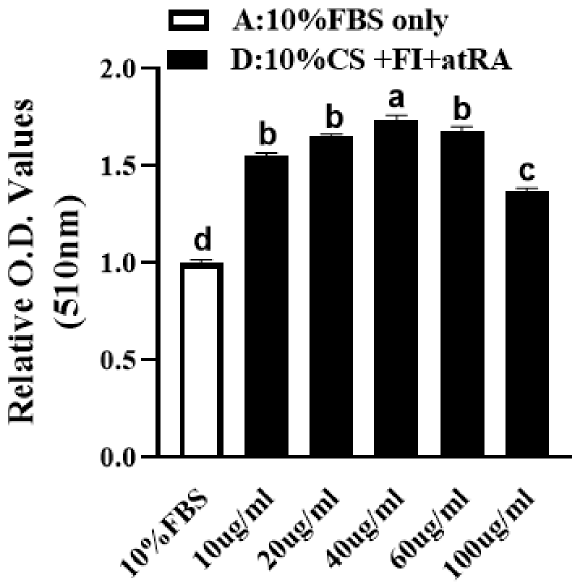

Figure S5. Induction effect of culture media containing different concentrations of atRA.

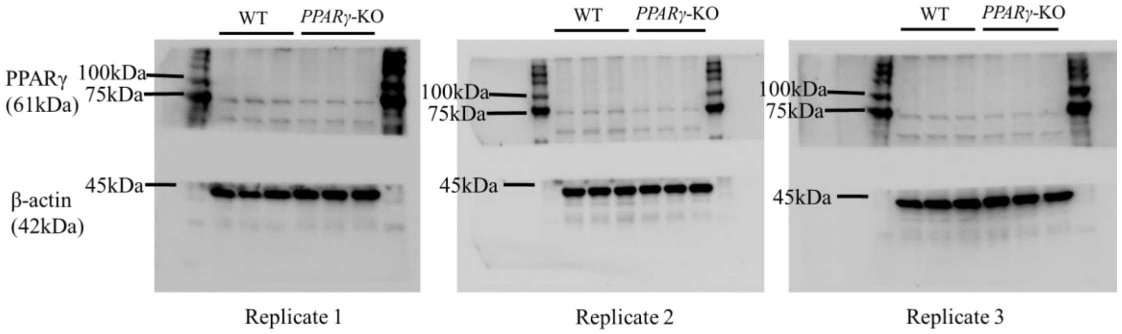

Figure S6. Western blot images that are uncropped and include a protein marker.
